# Complete mitochondrial genome of *Rectorisluxiensis* (Teleostei, Cyprinidae): characterisation and phylogenetic implications

**DOI:** 10.3897/BDJ.11.e96066

**Published:** 2023-01-10

**Authors:** Mingyao Zhang, Qiang Zhou, Hongmei Xiang, Jinxiu Wang, Xiangying Lan, Qinghua Luo, Wansheng Jiang

**Affiliations:** 1 Hunan Engineering Laboratory for Chinese Giant Salamander’s Resource Protection and Comprehensive Utilization, and Key Laboratory of Hunan Forest Products and Chemical Industry Engineering, Jishou University, Zhangjiajie, China Hunan Engineering Laboratory for Chinese Giant Salamander’s Resource Protection and Comprehensive Utilization, and Key Laboratory of Hunan Forest Products and Chemical Industry Engineering, Jishou University Zhangjiajie China; 2 College of Biology and Environmental Sciences, Jishou University, Jishou, China College of Biology and Environmental Sciences, Jishou University Jishou China

**Keywords:** Labeoninae, mitogenome assembly, annotation, phylogenetic relationship, evolution analysis

## Abstract

Mitochondrial genomes (mitogenomes) are widely used in scientific studies on phylogenetic relationships, molecular evolution and population genetics. Here, we sequenced and analysed the mitogenome of *Rectorisluxiensis*, a Yangtze River drainage endemic, but threatened cyprinid fish of Labeoninae. The complete mitogenome of *R.luxiensis* was 16,592 bp in length, encoding 13 protein coding genes (PCGs), 22 transfer RNA genes (tRNAs), two ribosomal RNA genes (rRNAs) and a control region. The mitogenome showed a high A+T content (58.2%) and a positive AT-skew (0.10) and negative GC-skew (–0.25) base composition pattern. All the 13 PCGs were found to start with ATG codons, except for the *COXI*, in which GTG was the start codon. The ratio of non-synonymous and synonymous substitutions (Ka/Ks) of all the 13 PCGs were less than 1, indicating negative or purifying selection evolved in these genes. Comparatively speaking, the evolutionary rate of *ATP8* was the fastest and *ND4L* was the slowest. All tRNAs could fold into a typical cloverleaf secondary structure, except tRNA^Ser1^ that lacked a dihydrouridine arm. Phylogenetic relationships, based on the PCGs dataset of 91 mitogenomes of Labeoninae, showed that *R.luxiensis* grouped with *Rectorisposehensis* and they formed a monophyletic *Rectoris*. However, many non-monophyletic genera were revealed in labeoninae fishes, such as *Cirrhinus*, *Decorus*, *Garra*, *Labeo* and *Pseudocrossocheilus*, which indicated that the validities of some traditional genera required a further check. This study reported the complete mitogenome of *R.luxiensis* for the first time, which provided valuable data for future molecular evolution and conservation related studies of *Rectoris* and other species in Labeoninae.

## Introduction

*Rectorisluxiensis* is a small-sized freshwater fish species that belongs to the Cyprinidae family in Cypriniformes. It has been recorded to distribute, endemically, only in some tributaries of the Yangtze drainage, including Yuanshui River and Xiangjiang River in the Hunan Province, Qingjiang River in the Hubei Province and Daning River in the Sichuan Province (Yue 2000). In morphology, *R.luxiensis* presents a typically modified structure of lip and jaw; thus, it has been categorised into the traditional Labeoninae, which was one of the twelve broadly recognised subfamilies in Cyprinidae (Yue 2000, Nelson et al. 2016, Jiang et al. 2018, Wang et al. 2022). Although recent phylogenetic studies showed that the Labeoninae would be modified as a tribe (Labeonini) in the subfamily Cyprininae (Yang et al. 2012, Yang et al. 2015), we tentatively kept it as a traditional subfamily here just for convenience. The species of Labeoninae show fantastic diversity in forms of lip and jaw, as well as other mouth-related structures (or oromandibular structures), which has been assumed to be able to settle into the turbulent water flow and scrape off the algae on the benthic substrate. Amongst many genera in Labeoninae, *Rectoris* is distinguished by the following combination of characteristics: upper lip absent, rostral cap developed and covering upper jaw completely, upper jaw linked to lower lip by a frenum, premaxillary barbells not well-developed, without mental grooves and lower lip not modified into adhesive disc (Yue 2000, Zheng et al. 2016). Although the *Rectoris* fishes are small-sized that usually grow up to less than 20 cm (Yue 2000), many of them are still under threats because of their capture for meat, especially for its delicate flavour from its relatively high contents of lipids. For this reason, it has usually been called an “oil fish” in many local areas.

During earlier times, new genera or species identifications and classifications amongst Labeoninae were merely from morphological studies (e.g. Zheng et al. (2010a), Huang et al. 2014); however, it was usually challenging while facing a wide variety of the oromandibular structures within the group. After all, Labeoninae is now one of the most diverse subfamilies of Cyprinidae that have about 40 genera and 400 species found from Asia to Africa (Wang et al. 2022) or even more according to the catalogue of fishes (Fricke et al. 2022). With the help of molecular data, more and more new taxa have been identified recently by using morphological comparisons along with phylogenetic inferences (e.g. Yao et al. (2018), Wang et al. (2022)). Given a lot of species in Labeoninae are still largely unknown since they have been described initially, obtaining more and more molecular data would be crucial for further understanding the phylogenetic relationships and evolutionary history of these species.

Vertebrate mitochondrial genome (mitogenome) is double-stranded circular DNA with typically 15 ~ 18 kb in length, with many characteristics like maternal inheritance, stable genetic components, fast evolutionary rate, low recombination frequency and highly conserved coding regions (Wolstenholme 1992, Boore 1999). Mitochondrial DNAs (mtDNAs), as molecular markers for evolutionary phylogenetics and population genetics, have been extensively used in a variety of species (Simon and Hadrys 2013, Sun et al. 2016, Hao et al. 2021, Zhao et al. 2021). As an endemic, but threatened fish of Labeoninae in the Yangtze River drainage, the *R.luxiensis*, received very little attention and few scientific studies since it has been described in 1977. The current knowledge of this species in science has been mainly restricted to the original morphological descriptions and distributions, whereas seldom further phylogenetic studies have been involved with it, not to mention its population structure and genetic diversity. In this study, we sequenced, assembled, annotated and reported the complete mitogenome of *R.luxiensis* for the first time, by which we aimed to promote the future studies of phylogenetics, population genetics and conservation biology of this species and other fishes in Labeoninae.

## Materials and methods

### Sample collection and sequencing

**Figure 1.**
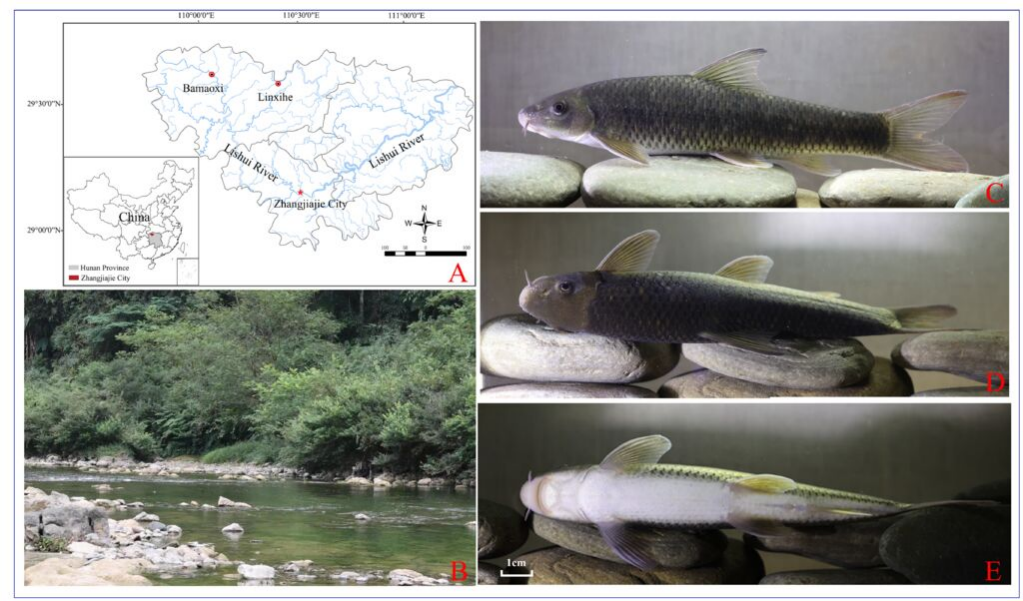
Sampling localities (A), typical habitat in Bamaoxi (B) and photos of a living specimen of *R.luxiensis* (C, lateral view; D, dorsal view; E, ventral view).

Samples of *R.luxiensis* were collected from two localities in the Lishui River drainage in Sangzhi County, Zhangjiajie City, Hunan Province of China (Fig. 1). One was collected from Linxihe (29°59′N, 110°29′E, n = 4) in September 2021 and the other was from Bamaoxi (29°63′N, 110°02′E, n = 13) in September 2022. All the samples were obtained by courtesy of local men through recreational fishing that was restricted to “one person with one rod and one fishhook”, which was allowed according to local laws. All specimens were preserved in 95% ethanol and deposited in the Engineering Laboratory at Jishou University. A unilateral pectoral fin from one sample in Linxihe (voucher No. JWS20210646) was cut out for DNA extraction by using the DNeasy Blood & Tissue Kit (Qiagen, Hilden, Germany). The DNA library was constructed and high-throughput sequencing was then conducted in paired-end mode on the DNBSEQ-T7 platform (Complete Genomics and MGI Tech, Shenzhen, China). Approximately 20 Gb of raw reads of 150 bp read length were generated.

### Mitogenome assembly and annotation

The complete mitogenome of *R.luxiensis* was assembled using NOVOPlasty 4.3 under default settings (Dierckxsens et al. 2017), with the mitogenome of *Rectorisposehensis* as a reference. The mitogenome annotation, including the preliminary location of protein-coding genes (PCGs) and ribosomal RNA genes (rRNAs) and the prediction of transfer RNA genes (tRNAs) were carried out by the MITOS web server (available at http://mitos2.bioinf.uni-leipzig.de/index.py, Donath et al. (2019)). The secondary structures of tRNAs were further identified using tRNAscan-SE Search Server (available at http://lowelab.ucsc.edu/tRNAscan-SE//, Lowe and Chan (2016)). The web application GeSeq was then employed to check up all the annotations and map the cycled mitogenome structure (available at https://chlorobox.mpimp-golm.mpg.de/geseq.html, Michael et al. (2017)). The final complete mitogenome sequence with annotation information of *R.luxiensis* has been submitted into NCBI (GenBank accession number: OP132373).

### Mitogenome characteristic analyses

The base structure, nucleotide composition and relative synonymous codon usage (RSCU) of different gene fragments were calculated using MEGA 11.0 (Tamura et al. 2021). The skewing of the nucleotide composition was calculated with the formulae: AT-skew = (A-T)/(A+T) and GC-skew = (G-C)/(G+C) (Perna and Kocher 1995). The ratio of non-synonymous substitutions (Ka) and synonymous substitutions (Ks) of each PCG were calculated by DNASP 6.0 (Rozas et al. 2017) for checking the signals of selective pressure, while Ka/Ks value > 1 indicates positive selection, Ka/Ks = 1 indicates neutral selection and Ka/Ks < 1 indicates negative or purifying selection. The plots of codons usage frequencies and Ka/Ks ratio were drawn using the Origin software (Moberly et al. 2018).

### Phylogenetic analyses

Mitogenomic sequences of 91 Labeoninae species, including the *R.luxiensis* which we sequenced in this study, were used for phylogenetic analyses, whereas a loach species *Cobitistakatsuensis* was selected as the outgroup. All the 13 PCGs were extracted and checked manually through MEGA 11.0 and then aligned using the in-built CLUSTALW algorithm with default settings (Tamura et al. 2021). The best-fit partitioning scheme and partition-specific models were calculated using Partitionfinder 2.1.1 (Lanfear et al. 2017), while the specific codons were assigned for each PCG. Phylogenetic analyses were conducted by both Maximum Likelihood (ML) and Bayesian Inference (BI) methods, based only on the PCGs dataset. The ML analysis was run in RaxML 8.0.2 (Stamatakis 2015) using the GTRGAMMA model and the best codon partition scheme, with the execution of 10 runs of random additional sequences and generating the bootstrap values following 1,000 rapid bootstrap replicates. The BI analysis was performed in MrBayes v.3.2.2 (Ronquist et al. 2012) by running 1.0×10^7^ million generations, while the posterior probabilities were calculated simultaneously with sampling every 1000 generations and discarding the initial 25% generations as burn-in.

## Results

### Mitogenomic structure and composition

**Figure 2.**
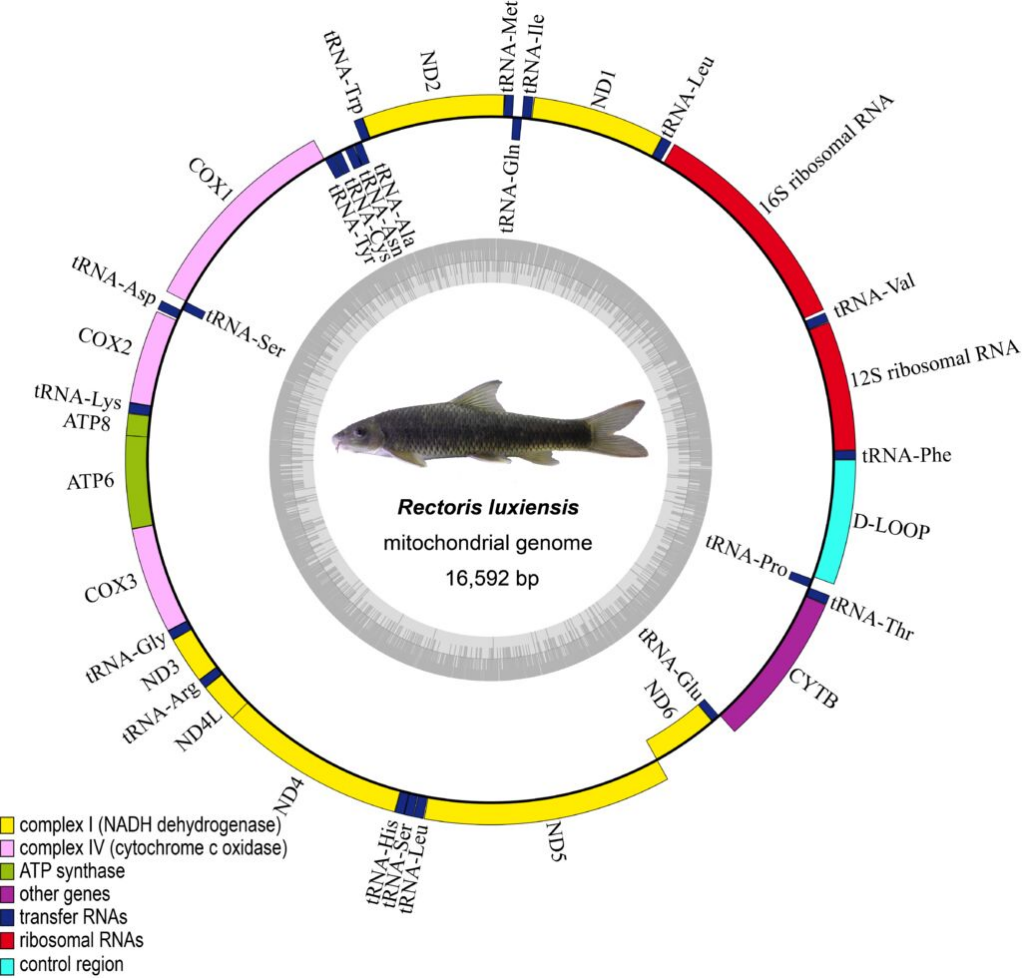
Circular gene map of the newly-sequenced mitogenome of *R.luxiensis*.

The complete mitogenome of *R.luxiensis* had a total length of 16,592 bp, which consisted of 13 typical vertebrate PCGs, 2rRNAs, 22 tRNAs and a non-coding control region (D-LOOP) (Fig. 2, Table 1). Amongst these genes, only one PCG (*ND6*) and eight tRNA genes (tRNA^Gln^, tRNA^Ala^, tRNA^Asn^, tRNA^Cys^, tRNA^Tyr,^ tRNA^Ser^, tRNA^Glu^ and tRNA^Pro^) were encoded by the L-strand and the remaining gene sequences were encoded by the H-strand. The mitogenome of *R.luxiensis* was compact, with ten gene overlaps, ranging from 1 to 7 bp in length. In addition, there were seventeen intergenic nucleotides (IGN) regions ranging from 1 to 24 bp in length and occupying a total of 84 bp, where the longest IGN (24 bp) was located between the tRNA^Val^ and 16S rRNA genes (Table 1).

The base compositions of *R.luxiensis* were A (32.0%) > C (26.2%) = T (26.2%) > G (15.7%), having a bias towards A+T (58.2%) in the complete mitogenome. The A+T bias also existed while looking at the A+T contents of PCGs (58.5%), rRNAs (54.9%), tRNAs (56.0%) and D-LOOP (67.3%), respectively (Table 2). The nucleotide compositions also showed a clear A and C bias pattern (AT-skew = 0.10, GC skew = –0.25), indicating a greater abundance of A than T and C than G (Table 2). Moreover, the AT skew and GC skew values of all the 13 PCGs were 0.02 and – 0.28, respectively, which indicated that the A and C bias pattern generally existed amongst the PCGs (Table 2).

### Characteristics of rRNAs and tRNAs

The two rRNAs (12S and 16S rRNA) were positioned between tRNA^phe^ and tRNA^leu^ and separated by tRNA^val^ in the mitogenome of *R.luxiensis*. The 12S rRNA was composed of 953 bp and the 16S rRNA was 1,640 bp in length. Both rRNA genes were encoded on the H-strand and displayed a positive AT skew and a negative GC skew (AT skew = 0.28, GC skew = – 0.08) (Table 2).

**Figure 3.**
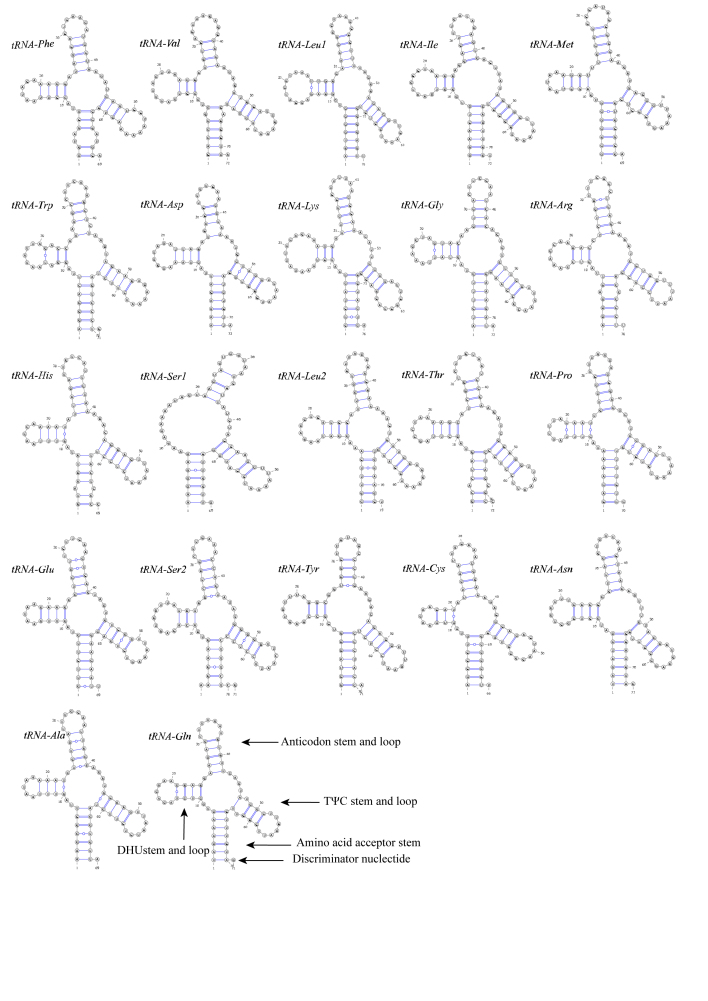
Secondary structure of 22 tRNA genes from the *R.luxiensis* mitogenome.

The mitogenome of *R.luxiensis* included 22 tRNAs as that in most vertebrates. The length of individual tRNA ranged from 66 to 76 bp and the concatenated total length of all tRNAs was 1,556 bp. The average AT skew was 0.11 and the average GC skew was –0.11 of these tRNAs, showing slightly higher A and C than T and G accordingly (Table 2). All tRNAs fold into typical cloverleaf secondary structures, except the tRNA^Ser1^ that lacked the dihydrouridine (DHU) arm (both stem and loop) (Fig. 3). In addition to the typical base pairs (G-C and A-U), there were also some wobble G-U pairs in the secondary structures of these tRNAs, which could also form stable chemical bonds between G and U. For instance, eight tRNAs (tRNA^Met^, tRNA^Lys^, tRNA^Ser1^, tRNA^Leu2^, tRNA^Glu^, tRNA^Ser2^, tRNA^Ala^ and tRNA^Cys^) showed G-U wobble base pairs in the acceptor stem, while another five (tRNA^Arg^, tRNA^Glu^, tRNA^Ser2^, tRNA^Tyr^ and tRNA^Ala^) in the anticodon stem. Additionally, five tRNAs (tRNA^Phe^, tRNA^Val^, tRNA^Arg^, tRNA^His^ and tRNA^Thr^) showed mismatched base pairs in the acceptor stem and three (tRNA^Trp^, tRNA^Asp^ and tRNA^Ser1^) in the anticodon stem.

### Characteristics of PCGs and codon usages

In the mitogenome of *R.luxiensis*, the PCGs comprised a concatenated length of 11,394 bp that accounted for 67.21% of the total sequence. All the 13 PCGs encoded on the H-strand, except *ND6* that was encoded by the L-strand. All the PCGs began with the regular start codon ATG, except that the *COX1* gene started with GTG. Ten PCGs were terminated with the conventional stop codons (TAA or TAG), while the other three (*ND4*, *COX2* and *CYTB*) were terminated with incomplete stop codons (TA or T) (Table 1).

**Figure 4.**
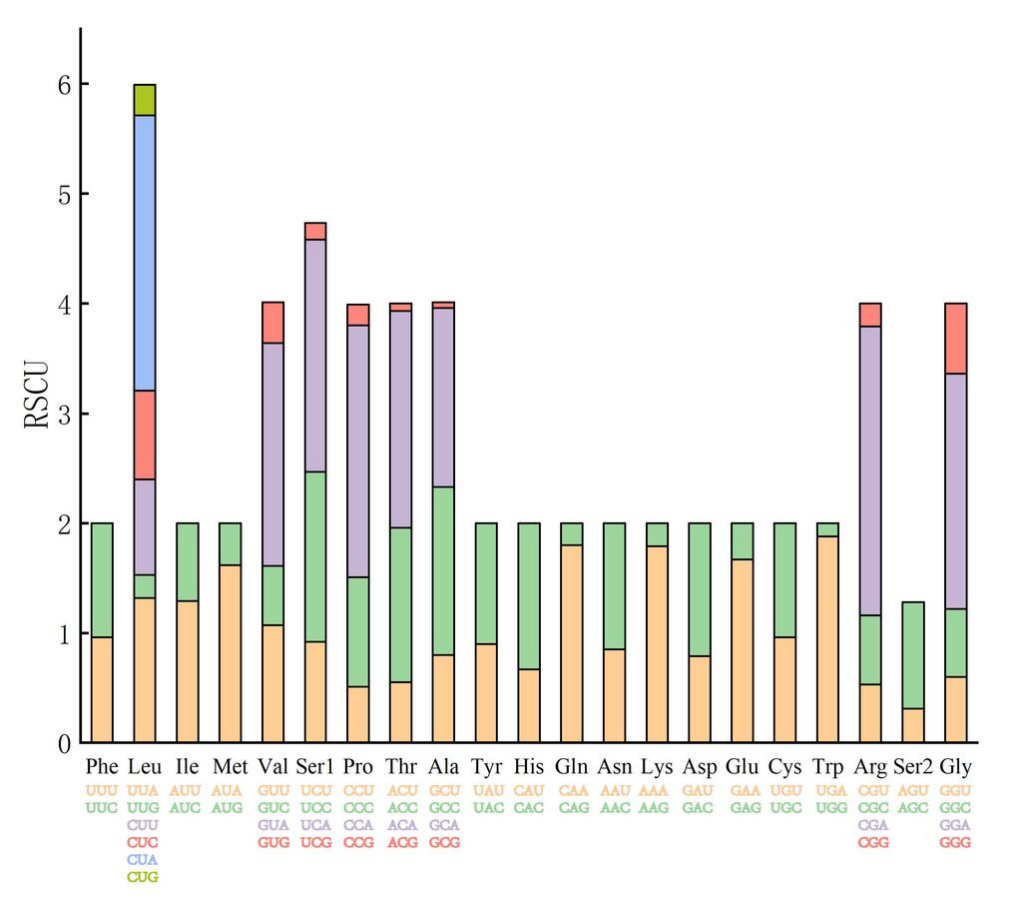
The relative synonymous codon usage (RSCU) in the mitogenome of *R.luxiensis*.

The RSCU values, based on 13 PCGs, showed that Leu encoded by the greatest number of synonymous codons (n = 6), while others were fewer: the Val, Ser1, Pro, Thr, Ala, Arg and Gly, were encoded by four codons and all the rest of the amino acids were encoded by only two codons (Fig. 4). While looking at each codon, the top three frequently-used codons of the PCGs were CGA (2.63%) encoded for Arg, CUA (2.5%) for Leu and CCA (2.29%) for Pro.

**Figure 5.**
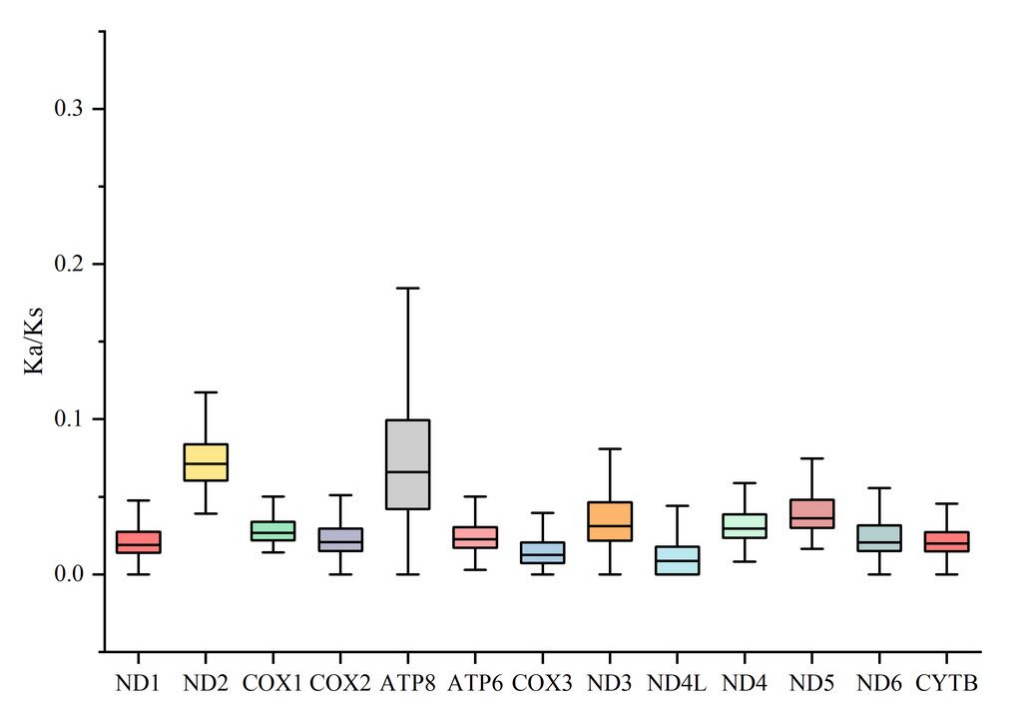
The Ka/Ks ratio of 13 PCGs amongst 91 species of Labeoninae.

The Ka/Ks ratios of the 13 PCGs, based on 91 species of Labeoninae, were all less than 1, with the highest Ka/Ks ratio in *ATP8* and the lowest ratio in *ND4L* (Fig. 5). None of the PCGs showed Ka/Ks ≥ 1 indicating a generally negative or purifying selection. Therefore, the evolution pattern of the mitogenome of Labeoninae tended to be conservative to maintain the regular functions of the generated proteins.

#### Phylogenetic analysis

**Figure 6.**
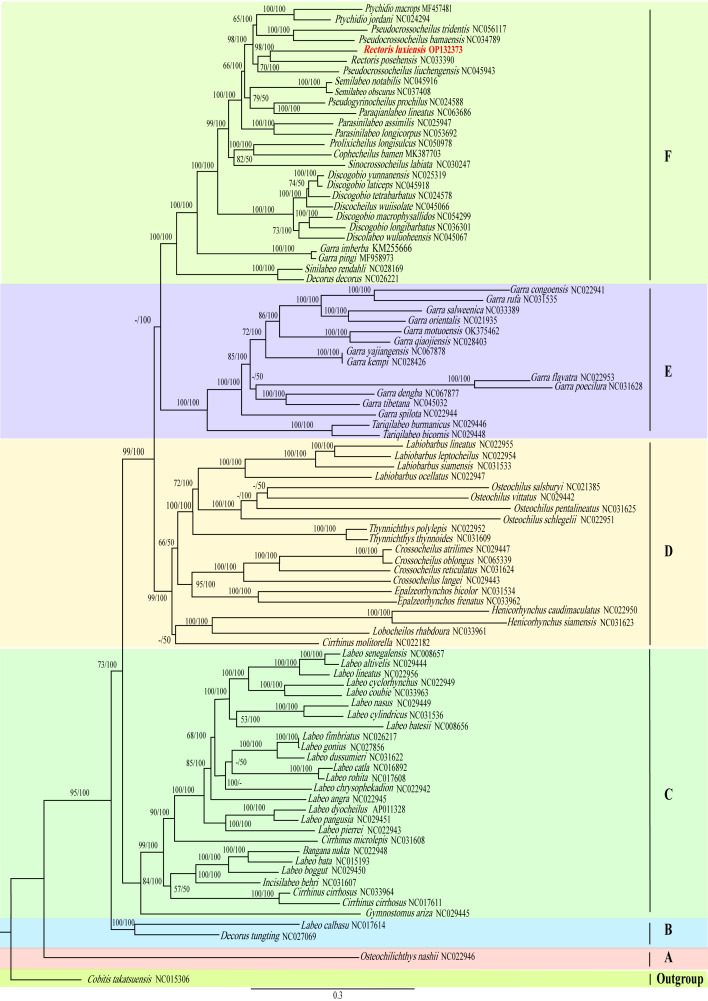
Phylogenetic relationship obtained from the ML method, based on 13 PCGs. Note: the numbers on the branches indicate bootstrap values from ML and posterior probabilities from the BI method. The GenBank accession number of each species is given in the brackets after the name. Red highlights the phylogenetic position of *R.luxiensis* that we obtained in this study.

Phylogenetic analyses were conducted, based on the 13 concatenated PCGs dataset from 91 species of Labeoninae (including *R.luxiensis* which we obtained in this study), while the *Cobitistakatsuensis* from the Cobitidae was used as the outgroup. Both BI and ML analyses generated trees with almost the same topologies, in which six major clades (here named clades A-G) could be distinguished (Fig. 6). Clade A, which included only one species in genus *Osteochilichthys*, diverged first. Then it was followed by clade B, which consisted of two species in *Labeo* and *Decorus*. The vast majority of species belonged to the remaining clades. Clade C included species from the following five genera: *Labeo*, *Cirrhinus*, *Bangana*, *Gymnostomus* and *Incisilabeo*. Although most of the species in *Labeo* were in this clade, they did not form a monophyletic group. Clade D included species in the following genera: *Lobocheilos*, *Henicorhynchus*, *Epalzeorhynchos*, *Crossocheilus*, *Thynnichthys* and *Osteochilus*. Clade E included species mainly in *Garra* and *Tariqilabeo*. Clade F contained the most number of genera, such as: *Garra*, *Semilabeo*, *Parasinilabeo*, *Prolixicheilu*, *Rectoris*, *Ptychidio*, *Pseudocrossocheilus*, *Sinocrossocheilus*, *Decorus*, *Discogobio*, *Paraqianlabeo*, *Cophecheilu* and *Pseudogyrinocheilus*. Although many of the genera were modified and reclassified, based on recent studies and we updated all the names according to the catalogue of fishes (up to Dec 2022), there were obviously many genera which were non-monophyletic according to this phylogenetic tree (Fig. 6). However, *R.luxiensis* appeared in clade F with a sister-group species to *R.posehensis* and the two species supported a monophyletic *Rectoris*.

## Discussion

We successfully sequenced and assembled the mitogenome of *R.luxiensis*, an endemic, but threatened fish of Labeoninae in the Yangtze River drainage, for the first time in this study. The mitogenome of *R.luxiensis* was 16,592 bp in length, which was similar to other known species of Labeoninae, such as 16,594 bp in *Rectorisposehensis*, 16,599 bp in *Semilabeonotabilis* and 16,600 bp in *Pseudocrossocheilusliuchengensis* (Wu et al. 2018, Liu et al. 2018). The slight length variations of mitogenomes of closely-related species usually resulted from the changes of tandem repeats within the control region and the lengths of intergenic regions or gene overlaps (Wang et al. 2020). Some characteristics of the mitogenome of *R.luxiensis* were also typical and similar to other Labeoninae fishes, including the contents and orders of 13 PCGs, 2rRNAs, 22 tRNAs and a D-LOOP and the encoding location for most genes was on the H-strand, with and only the *ND6* gene and eight tRNAs on the L-strand (Wu et al. 2018, Liu et al. 2018).

The nucleotide compositions and codon usages of mitogenomes of Cyprinidae were generally similar, but some detectable differences remained. For example, in *R.luxiensis*, only the *COX1* gene started with GTG, but for *Rhodeuscyanorostris*, both *ND1* and *COX1* genes started with GTG (Li et al. 2022). Whether the diverse usage of start codons amongst different species was generated randomly or with some evolutionarily meaningful preferences, this was an interesting question of selection, but without being given much attention. In addition, the secondary structure of tRNAs of *R.luxiensis* was overall conservative of vertebrate mitogenomes by having typical Watson-Crick pairings (G-C and A-U) (Wolstenholme 1992); however, there were still dozens of non-typical forms such as UG pairing in some different stem regions (Fig. 3), which was also revealed from other studies (e.g. Zhao et al. (2021)). Recent studies have indicated that tRNAs matched with non-typical pairings could also convert into fully functional proteins through post-transcriptional mechanisms (Chao et al. 2008, Pons et al. 2014).

Mitochondrial DNA sequences are widely used in phylogenetic studies (Sun et al. 2017, Tang et al. 2018, Chang et al. 2020). It has been generally recognised that the complete mitogenome could uncover evolutionary relationships better than individual mitochondrial genes (Hou et al. 2020). In this study, a phylogenetic hypothesis of Labeoninae was able to be reconstructed while using the mitogenomes of 91 representative species, including the *R.luxiensis* which we sequenced in this study. Previous studies have shown that the monophyly of Labeoninae was supported, but the inter-generic relationships were usually controversial (e.g. Li et al. (2005), Wang et al. (2020)). Our study suggested that some previous inconsistences may result from the limited phylogenetic information from less gene fragments. For instance, the sister group relationship of *Rectoris* and *Pseudocrossocheilus* in our study was consistent with a previous study that was also based on mitogenomes (Wang and Zeng 2021), but this relationship was not revealed from the study from a single 16S rRNA gene (Li et al. 2005). We found that the phylogenetic trees of the mitochondrial and nuclear genes in Labeoninae were sometimes different, possibly due to the different evolutionary rates and variable informative sites (Lynch et al. 2006). For example, *Rectoris* and *Semilabeo* were once revealed as a sister group relationship, based on two nuclear genes (Zheng et al. 2010b), which was also inconsistent with our study (Fig. 6).

In addition, our study also suggested that some inconsistent inter-generic relationships within Labeoninae might be from the non-monophyletic nature of some traditionally recognised genera (mostly from morphological hypotheses), such as *Cirrhinus*, *Decorus*, *Garra*, *Labeo* and *Pseudocrossocheilus* which we detected in this study (Fig. 6). Thus, future studies should pay more attention to these genera. Let us take *Garra*, a traditionally large group that was erected as early as 1822, as an example. It has usually served as a taxonomic wastebasket for species having disc on the lower lip but could not be assigned into other genera. Recently, some new genera have been identified and separated from *Garra*-like species, such as *Sinigarra* (Zhang et al. 2012) and *Guigarra* (Wang et al. 2022). Although with some of the inconsistent intergeneric relationships, the genera included in each major clade were largely similar with previous studies (Zheng et al. 2010b, Yang et al. 2012, Li and Han 2018, Pan et al. 2020). In brief, the phylogenetic relationships within Labeoninae are still far from resolved; thus, more and more molecular data, such as the one we reported in this study, will be necessary and helpful for understanding the evolutionary history and diversity of this complicated, but fantastic group.

## Data resources

The genome sequence data are available in GenBank (https://www.ncbi.nlm.nih.gov/) under accession no. OP132373.

## Funding

This work was supported in part by the Innovation Platform and Talent Plan of Hunan Province [2020RC3057]; the National Natural Science Foundation of China [32060128]; Zhilan foundation [2020040371B, 2022010011B] and opening projects of Hunan Engineering Laboratory for Chinese Giant Salamander’s Resource Protection and Comprehensive Utilization [DNGC2211].

## Conflicts of interest

No potential conflict of interest was reported by the author(s).

## Figures and Tables

**Table 1.** Table 1 Characteristics of the mitogenome of *R.luxiensis*.

Gene	Position		Length	Codon		Anticodon	Intergenic nucleotides*	Strand
	From	To		Start	Stop			
tRNA^Phe^	1	69	69			GAA		H
12S rRNA	70	1,022	953				2	H
tRNA^Val^	1,025	1,096	72			TAC	20	H
16S rRNA	1,117	2,756	1,640				24	H
tRNA^Leu^	2,781	2,856	76			TAA	1	H
*ND1*	2,858	3,832	975	ATG	TAA		4	H
tRNA^Ile^	3,837	3,908	72			GAT	–2	H
tRNA^Gln^	3,907	3,977	71			TTG	1	L
tRNA^Met^	3,979	4,047	69			CAT	0	H
*ND2*	4,048	5,094	1,047	ATG	TAG		–2	H
tRNA^trp^	5,093	5,163	71			TCA	2	H
tRNA^Ala^	5,166	5,234	69			TGC	1	L
tRNA^Asn^	5,236	5,308	73			GTT	2	L
NCR	5,311	5,342	32				–1	H
tRNA^Cys^	5,342	5,407	66			GCA	1	L
tRNA^Tyr^	5,409	5,479	71			GTA	1	L
*COX1*	5,481	7,031	1,551	GTG	TAA		0	H
tRNA^Ser^	7,032	7,102	71			TGA	3	L
tRNA^Asp^	7,106	7,177	72			GTC	13	H
*COX2*	7,191	7,881	691	ATG	T--		0	H
tRNA^Lys^	7,882	7,957	76			TTT	1	H
*ATP8*	7,959	8,123	165	ATG	TAA		–7	H
*ATP6*	8,117	8,800	684	ATG	TAA		–1	H
*COX3*	8,800	9,585	786	ATG	TAA		–1	H
tRNA^Gly^	9,585	9,656	72			TCC	0	H
*ND3*	9,657	10,007	351	ATG	TAG		–2	H
tRNA^Arg^	10,006	10,075	70			TCG	0	H
*ND4L*	10,076	10,372	297	ATG	TAA		–7	H
*ND4*	10,366	11,743	1,378	ATG	T--		0	H
tRNA^His^	11,744	11,812	69			GTG	0	H
tRNA^Ser^	11,813	11,881	67			GCT	1	H
tRNA^Leu^	11,883	11,955	73			TAG	3	H
*ND5*	11,959	13,782	1,824	ATG	TAA		–4	H
*ND6*	13,779	14,300	522	ATG	TAG		0	L
tRNA^Glu^	14,301	14,369	69			TTC	4	L
*CYTB*	14,374	15,514	1,141	ATG	T--		0	H
tRNA^Thr^	15,515	15,586	72			TGT	–1	H
tRNA^Pro^	15,586	15,655	70			TGG	16	L
D-LOOP	15,672	16,592	921				0	H
Notes: * The numbers of nucleotides between the given and its previous gene, negative values indicate an overlap; T-- indicated incomplete stop codon; H and L indicated that the genes are transcribed on the heavy and light strand, respectively.								

**Table 2.** Nucleotide composition (in percentages) and skew of the mitogenome of *R.luxiensis*.

gene	size（bp）	T%	C%	A%	G%	A+T%	C+G%	AT-skew	GC-skew
*ND1*	972	26.3	28.8	30.5	14.4	56.8	43.2	0.07	–0.33
*ND2*	1,044	24.2	30.8	32.8	12.2	57.0	43.0	0.15	–0.43
*COX1*	1,548	29.8	25.3	27.8	17.0	57.6	42.3	–0.03	–0.20
*COX2*	690	27.1	25.8	31.4	15.7	58.5	41.5	0.07	–0.24
*ATP8*	165	26.1	26.7	36.4	10.9	62.5	37.6	0.16	–0.42
*ATP6*	684	30.0	26.5	30.7	12.7	60.7	39.2	0.01	–0.35
*COX3*	783	26.7	28.5	28.6	16.2	55.3	44.7	0.03	–0.28
*ND3*	348	39.3	28.7	27.6	14.4	66.9	43.1	–0.17	–0.33
*ND4L*	294	27.2	30.6	26.5	15.6	53.7	46.2	–0.01	–0.32
*ND4*	1,380	27.2	26.9	32.1	13.7	59.3	40.6	0.08	–0.33
*ND5*	1,821	28.5	26.1	32.9	12.5	61.4	38.6	0.07	–0.35
*ND6*	519	42.8	11.6	13.5	32.2	56.3	43.8	–0.52	0.47
*CYTB*	1,140	29.6	26.3	29.8	14.2	59.4	40.5	0.00	–0.30
rRNAs	2,593	19.7	24.4	35.2	20.7	54.9	45.1	0.28	–0.08
tRNAs	1,556	24.9	24.5	31.1	19.5	56.0	44.0	0.11	–0.11
D-LOOP	921	33.6	19.1	33.7	13.7	67.3	32.8	0.00	–0.16
PCGs	11,394	28.6	26.5	29.9	15.0	58.5	41.5	0.02	–0.28
Total	16,952	26.2	26.2	32.0	15.7	58.2	41.9	0.10	–0.25
